# Association of rs5888 SNP in the scavenger receptor class B type 1 gene and serum lipid levels

**DOI:** 10.1186/1476-511X-11-50

**Published:** 2012-07-09

**Authors:** Dong-Feng Wu, Rui-Xing Yin, Xi-Jiang Hu, Lynn Htet Htet Aung, Xiao-Li Cao, Lin Miao, Qing Li, Ting-Ting Yan, Jin-Zhen Wu, Shang-Ling Pan

**Affiliations:** 1 Department of Cardiology, Institute of Cardiovascular Diseases, the First Affiliated Hospital, Guangxi Medical University, 22 Shuangyong Road, Nanning, 530021, Guangxi, People’s Republic of China; 2 Department of Pathophysiology, School of Premedical Sciences, Guangxi Medical University, Nanning, 530021, Guangxi, People’s Republic of China

## Abstract

**Background:**

Bai Ku Yao is a special subgroup of the Yao minority in China. The present study was undertaken to detect the association of rs5888 single nucleotide polymorphism (SNP) in the scavenger receptor class B type 1 (SCARB1) gene and several environmental factors with serum lipid levels in the Guangxi Bai Ku Yao and Han populations.

**Methods:**

A total of 598 subjects of Bai Ku Yao and 585 subjects of Han Chinese were randomly selected from our stratified randomized cluster samples. Genotypes of the SCARB1 rs5888 SNP were determined by polymerase chain reaction and restriction fragment length polymorphism combined with gel electrophoresis, and then confirmed by direct sequencing.

**Results:**

The levels of total cholesterol (TC), high-density lipoprotein cholesterol (HDL-C), low-density lipoprotein cholesterol (LDL-C), apolipoprotein (Apo) AI were lower but ApoB was higher in Bai Ku Yao than in Han (*P* < 0.05-0.001). The frequencies of C and T alleles were 78.3% and 21.7% in Bai Ku Yao, and 73.7% and 26.3% in Han (*P* < 0.01); respectively. The frequencies of CC, CT and TT genotypes were 60.0%, 36.6% and 3.4% in Bai Ku Yao, and 54.2%, 39.0% and 6.8% in Han (*P* < 0.01); respectively. The subjects with TT genotype in both ethnic groups had lower HDL-C and ApoAI levels than the subjects with CC or CT genotype (*P* < 0.05 for all). Subgroup analyses showed that the subjects with TT genotype in Bai Ku Yao had lower HDL-C and ApoAI levels in males than the subjects with CC or CT genotype (*P* < 0.05 for all), and the T allele carriers had higher TC, LDL-C and ApoB levels in females than the T allele noncarriers (*P* < 0.05 for all). The participants with TT genotype in Han also had a lower tendency of HDL-C and ApoAI levels in males than the participants with CC or CT genotype, but the difference did not reach statistically significant (*P* = 0.063 and *P* = 0.086; respectively). The association of serum HDL-C and ApoAI levels and genotypes was confirmed by the multiple linear regression analysis in both ethnic groups. Serum lipid parameters were also correlated with several environmental factors.

**Conclusions:**

The differences in serum lipid levels between the two ethnic groups might partially attribute to the differences in the SCARB1 rs5888 SNP and several environmental factors.

## Introduction

Coronary heart disease (CHD) remains a major cause of worldwide morbidity and mortality despite therapeutic advances that control many risk factors such as low-density lipoprotein cholesterol (LDL-C) to levels lower than previously possible [1]. Plasma high-density lipoprotein cholesterol (HDL-C) levels are considered as a major determinant of susceptibility to coronary atherosclerosis in the general population [2,3]. A low plasma HDL-C level is the most common lipid abnormality found in families with premature coronary atherosclerosis [2]. Epidemiological studies showed that plasma HDL-C levels are inversely associated with the risk of CHD: an increase of 1 mg/dl of HDL-C levels is associated with a 2% to 3% decrease of the risk for CHD [4,5]. The metabolism of HDL-C is complex, with many factors influencing its circulating plasma levels, both genetic and non-genetic. It has been reported that variation in HDL-C levels is at least 50% genetically determined [6]. A number of variants in candidate genes have been implicated in the regulation of plasma HDL-C levels [7,8].

The scavenger receptor class B type 1 (SCARB1) is the HDL receptor which binds HDL-C with high affinity. It is expressed primarily in liver and nonplacental steroidogenic tissues and mediates selective cholesterol uptake of HDL by a mechanism distinct from the classic LDL-C receptor pathway, and plays an important role in reverse cholesterol translation (RCT) [9]. In mouse models, studies have clearly demonstrated the crucial role of SCARB1 gene in HDL-C metabolism. Hepatic overexpression of SCARB1 markedly reduced HDL-C and apolipoprotein (Apo) AI levels, and increased biliary cholesterol [10]. In contrast, targeted disruption of the SCARB1 gene in mice reduced selective uptake of cholesterol ester from HDL into the liver and significantly increased plasma HDL-C [11,12]. As a multilipoprotein receptor, SCARB1 also regulates the concentrations of LDL-C and very-low-density lipoprotein (VLDL-C) [13,14]. Mice with hepatic overexpression of SCARB1 have lower concentrations of VLDL and LDL [15,16]. Conversely, mice with attenuated expression of SCARB1 display elevated concentration of LDL-C [17]. Despite the obvious functional evidence for an influence of SCARB1 on altered serum lipid profile in animal models, it remains to be determined whether this receptor has an equally important function in humans.

The human SCARB1 gene encodes a 509 amino acid protein with a molecular weight of 82 kDa [13] and located on chromosome 12q24, a region showing significant linkage to plasma HDL-C levels [18]. Previous studies have investigated the relationship between variants in the SCARB1 gene and alterations on serum lipid profile in diverse human populations [19-24]. The single nucleotide polymorphism (SNP) of rs5888, a“C” to “T” substitution at amino acid 350 in exon 8, has been associated with the lipid concentrations of HDL-C [19,21,23,24] and LDL-C [20,21,23]. The C allelic frequency of SCARB1 rs5888 was varied from 40% to 60% in white population [19-23]. However, in Korean subjects the C allelic frequency of SCARB1 rs5888 was 67% in control and 83% in CHD [24]. These studies suggest that the polymorphism of SCARB1 rs5888 might be different among diverse ethnic groups. Thus, the SCARB1 rs5888 polymorphism in other racial groups still needs to be determined [24].

China is a multi-ethnic country. There are 56 ethnic groups. Han is the largest ethnic group and Yao is the eleventh largest minority among the 55 minority groups according to the population size. Bai Ku Yao (White-trouser Yao), an isolated branch of the Yao minority, is named so because all of the men wear white knee-length knickerbockers. The population size is about 30000. Because of isolation from the other ethnic groups, the special customs and cultures including their clothing, intra-ethnic marriages, dietary habits, and corn wine and rum intakes are still completely preserved to the present day. They are currently in a transitional period from the matriarchal society to patriarchal society. In several previous epidemiological studies, we showed that several serum lipid phenotypes were lower in Bai Ku Yao than in Han Chinese from the same region [25,26]. This ethnic difference in serum lipid profiles is still not well known. We hypothesized that some genetic factors may be different between the two ethnic groups. The SNP of rs5888 in SCARB1 gene had not been discussed in the Chinese population. The aim of the present study was to determine the association of SCARB1 rs5888 SNP and several environmental factors with serum lipid levels in the Guangxi Bai Ku Yao and Han populations.

## Materials and methods

### Study population

This study included 598 subjects of Bai Ku Yao who reside in Lihu and Baxu villages in Nandan County, Guangxi Zhuang Autonomous Region, People’s Republic of China and 585 subjects of Han Chinese reside in the same villages. The subjects of Bai Ku Yao consisted of 286 (47.8%) males and 312 (52.2%) females, ranged in age from 16 to 80 years, with a mean age of 40.22 ± 15.18 years. The subjects of Han consisted of 277 (47.4%) males and 308 (52.6%) females, aged 16–92 years, with a mean age of 41.87 ± 16.35 years. All of the subjects were randomly selected from our previous stratified randomized cluster samples [25,26]. The study subjects were healthy rural agricultural workers and without evidence of any chronic illness, including hepatic, renal, or thyroid. The participants with a history of heart attack or myocardial infarction, stroke, congestive heart failure, diabetes have been excluded. The participants were not on any lipid-lowering treatment. The present study was approved by the Ethics Committee of the First Affiliated Hospital, Guangxi Medical University. Informed consent was obtained from all subjects after they received a full explanation of the study.

### Epidemiological survey

The survey was carried out using internationally standardized methods [27]. Information on demographics, socioeconomic status, and lifestyle factors were collected with standardized questionnaires. The alcohol information included questions about the number of liangs (about 50 g) of rice wine, corn wine, rum, beer, or liquor consumed during the preceding 12 months. At the physical examination, several parameters such as height, weight, and waist circumference were measured. Sitting blood pressure was measured using a mercury sphygmomanometer on 3 separated intervals after the subjects had a 5-minute rest, and the average of the three measurements was used for the level of blood pressure. Body mass index (BMI) was calculated as weight in kg divided by the square of height in meters (kg/m^2^).

### Biochemical analysis

Venous blood samples were obtained from all subjects after at least 12 hours of fasting. The levels of serum TC, triglyceride (TG), HDL-C, and LDL-C in samples were determined by enzymatic methods with commercially available kits. Serum ApoAI and ApoB levels were detected by the immunoturbidimetric immunoassay using a commercial kit [25,26] .

### DNA amplification and genotyping

Genomic DNA was extracted from peripheral blood leukocytes using the phenol-chloroform method [28]. Genotyping of the SCARB1 rs5888 SNP was performed by polymerase chain reaction and restriction fragment length polymorphism (PCR-RFLP). A pair of primers was designed to introduce a *Hin*I1 restriction site (GACGCC) by changing a base from A to G. Forward 5'-CCTTGTTTCTCTCCCATCCTCACTTCCTCGACGC-3', Reverse primer 5'-CACCACCCCAGCCCACAGCAGC-3' (Sangon, Shanghai, People’s Republic of China). Each 20 μL PCR reaction mixture consisted of 1 μL of genomic DNA, 0.5 μL of each primer (10 pmol/L), 10 μL of 2 × Taq PCR Mastermix (constituent: 20 mM Tris–HCl, pH 8.3, 100 mM KCl, 3 mM MgCl_2_, 0.1 U *Taq* Polymerase/μL, 500 μM dNTP each; Tiangen, Beijing, People’s Republic of China), and 8 μL of ddH_2_O (DNase/RNase-free). The reaction mixture was subjected to denaturation at 95°C for 5 min, followed by 33 cycles at 95°C for 45 s, 71.5°C for 30 s, 72°C for 50 s, then by a final extension at 72°C for 8 min. The quality of PCR products was controlled by electrophoresis on 2% agarose gel and visualized with ethidium-bromide staining ultraviolet illumination. Then 5 μL of amplification products were digested at 37°C overnight with 5 U of *Hin*I1 restriction enzyme (Fermentas Co. Canada). After restriction enzyme digestion of the amplified DNA, the fragments were separated by electrophoresis on 3% agarose gels stained with ethidium bromide, photographed in ultraviolet light. Genotypes were scored by an experienced reader blinded to epidemiological data and serum lipid levels. Six samples (CC, CT and TT genotypes in two; respectively) detected by the PCR-RFLP were also confirmed by direct sequencing.

### Diagnostic criteria

The normal values of serum TC, TG, HDL-C, LDL-C, ApoAI, ApoB levels, and the ratio of ApoAI to ApoB in our Clinical Science Experiment Center were 3.10-5.17, 0.56-1.70, 0.91-1.81, 2.70-3.20 mmol/L, 1.00-1.78, 0.63-1.14 g/L, and 1.00-2.50; respectively[25,26]. Hypertension was diagnosed according to the criteria of 1999 World Health Organization-International Society of Hypertension Guidelines for the management of hypertension [29]. Normal weight, overweight and obesity were defined as a BMI < 24, 24–28, and > 28 kg/m^2^; respectively [25,26].

### Statistical analyses

Epidemiological data were recorded on a pre-designed form and managed with Excel software. All statistical analyses were carried out using the statistical software package SPSS 13.0 (SPSS Inc., Chicago, Illinois). Qualitative variables were expressed as raw count and percentage. Mean ± standard deviation was used for the presentation of quantitative variables. Genotypic and allelic frequencies were calculated by direct counting, the Hardy-Weinberg equilibrium (HWE) was tested by chi-square, using the observed genotypic frequencies obtained from the data and expected genotypic frequency obtained using the HWE. A chi-square analysis was also used to evaluate the difference in genotype distribution and sex ratio between the groups. The difference in general characteristics between Bai Ku Yao and Han was tested by the Student’s unpaired *t*-test. The association of genotypes and serum lipid parameters was tested by analysis of covariance (ANCOVA). Sex, age, BMI, blood pressure, alcohol consumption, cigarette smoking were adjusted for the statistical analysis. All significant associations were corrected for multiple testing by applying a Bonferroni correction. In order to evaluate the association of serum lipid levels with genotypes (CC = 1, CT = 2, TT = 3) and several environment factors, multiple linear regression analysis with stepwise modeling was also performed in the combined population of Bai Ku Yao and Han, Bai Ku Yao, Han, males and females; respectively. A two-tailed *P* value less than 0.05 was considered statistically significant.

## Results

### General characteristics and serum lipid levels

The general characteristics and serum lipid levels between the Bai Ku Yao and Han populations are summarized in Table 1. As comparison with the population of Han, Bai Ku Yao has lower levels of height, weight, systolic blood pressure, diastolic blood pressure, serum TC, HDL-C, LDL-C, ApoAI, and higher serum ApoB levels, percentages of subjects who consumed alcohol or smoked cigarettes (*P <* 0.05-0.001). There were no significant differences in the levels of BMI, pulse pressure, serum TG, the ratio of ApoAI to ApoB, age structure, or the ratio of male to female between the two ethnic groups (*P* > 0.05 for all).

**Table 1:** The general characteristics and serum lipid levels

Parameter	Bai Ku Yao	Han Chinese	*t* (*χ*^2^)	*P*
Number	598	585	–	–
Male/female	286/312	277/308	0.027	0.870
Age (years)	40.22 ± 15.18	41.87 ± 16.35	−1.795	0.073
Height (cm)	152.64 ± 7.37	156.33 ± 7.90	−8.318	0.000
Weight (kg)	51.69 ± 7.23	54.57 ± 9.74	−5.742	0.000
Body mass index (kg/m^2^)	22.15 ± 2.38	22.29 ± 3.46	−0.814	0.416
Systolic blood pressure (mmHg)	119.26 ± 17.44	122.78 ± 18.76	−3.352	0.001
Diastolic blood pressure (mmHg)	75.46 ± 9.69	77.58 ± 11.52	−3.430	0.001
Pulse pressure (mmHg)	43.80 ± 13.23	45.20 ± 12.79	−1.855	0.064
Cigarette smoking [n (%)]				
Nonsmoker	414 (69.2)	437 (74.7)		
< 20 cigarettes/day	84 (14.0)	57 (9.7)		
≥ 20 cigarettes/day	100 (16.7)	91 (15.6)	6.074	0.048
Alcohol consumption [n (%)]				
Nondrinker	329 (55.0)	458 (78.3)		
< 25 g/day	172 (28.8)	80 (13.7)	71.959	0.000
≥ 25 g/day	97 (16.2)	47 (8.0)		
Total cholesterol (mmol/L)	4.35 ± 0.92	4.71 ± 0.99	−6.551	0.000
Triglyceride (mmol/L)	1.00 (0.65)	1.00 (0.79)	−1.734	0.083
HDL-C (mmol/L)	1.67 ± 0.42	1.73 ± 0.46	−2.431	0.015
LDL-C (mmol/L)	2.57 ± 0.76	2.72 ± 0.79	−3.502	0.000
Apolipoprotein (Apo) AI (g/L)	1.31 ± 0.32	1.35 ± 0.31	−2.110	0.035
ApoB (g/L)	0.85 ± 0.23	0.82 ± 0.20	2.314	0.021
ApoAI/ApoB	1.67 ± 0.75	1.73 ± 0.53	−1.382	0.167

HDL-C, high-density lipoprotein cholesterol; LDL-C, low-density lipoprotein cholesterol. The value of triglyceride was presented as median (interquartile range). The difference between the two ethnic groups was determined by the Wilcoxon-Mann–Whitney test.

### Results of electrophoresis and genotyping

**Figure 1:**
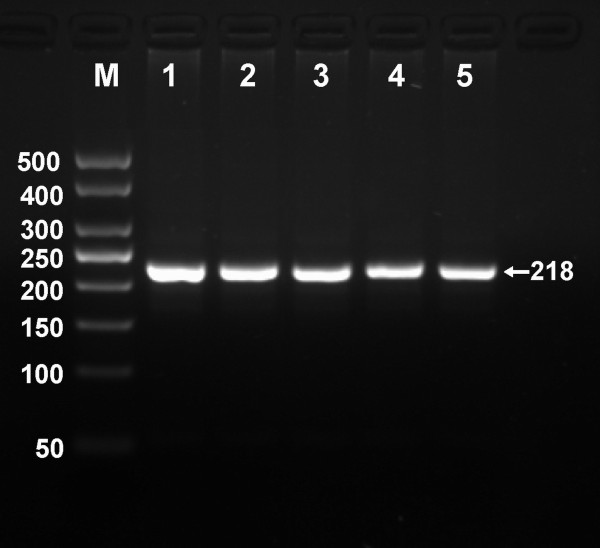
**Electrophoresis of PCR products of the samples.** Lane M, 50 bp marker ladder; lanes 1–5, samples. The 218 bp bands are the PCR products.

**Figure 2:**
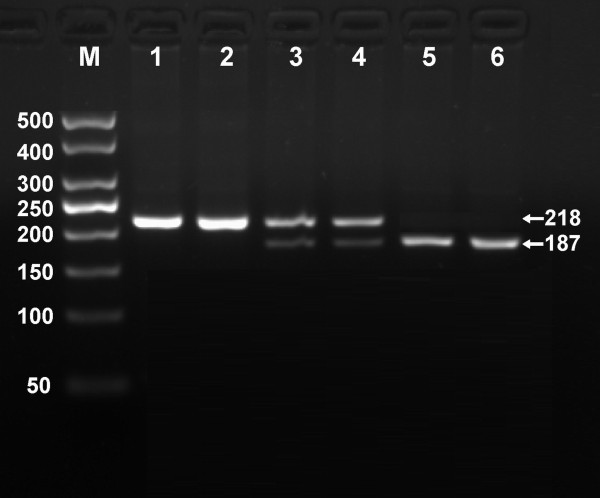
**Genotyping of rs5888 SNP in the SCARB1 gene.** Lane M, 50 bp marker ladder; lanes 1–2, TT genotype (218 bp); lanes 3 and 4, CT genotype (218-, 187- and 31-bp); and lanes 5 and 6, CC genotype (187- and 31-bp). The 31 bp fragment was invisible in the gel owing to its fast migration speed.

After the genomic DNA of the samples was amplified by PCR and imaged by 2.0% agarose gel electrophoresis, the PCR products of 218 bp nucleotide sequences could be found in all samples (Figure 1). The genotypes identified were named according to the presence or absence of the enzyme restriction sites, when a C to T transversion at amino acid 350 of the SCARB1 gene. The presence of the cutting site indicates the C allele, while its absence indicates the T allele (cannot be cut). Thus, the TT genotype is homozygote for the absence of the site (band at 218 bp), CT genotype is heterozygote for the absence and presence of the site (bands at 218-, 187- and 31-bp), and CC genotype is homozygote for the presence of the site (bands at 187- and 31-bp; Figure 2). The 31 bp fragment was invisible in the gel owing to its fast migration speed.

### Genotypic and allelic frequencies

The genotypic and allelic frequencies of SCARB1 rs5888 SNP are shown in Table 2. The frequency of C and T alleles was 78.3% and 21.7% in Bai Ku Yao, and 73.7% and 26.3% in Han (*P* < 0.01); respectively. The frequency of CC, CT and TT genotypes was 60.0%, 36.6% and 3.3% in Bai Ku Yao, and 54.2%, 39.0% and 6.8% in Han (*P* < 0.01); respectively. There was no significant difference in the genotypic and allelic frequencies between males and females in both ethnic groups.

**Table 2:** The genotype and allele frequencies of the SCARB1 rs5888 SNP [n (%)]

Group	n	Genotype			Allele	
		CC	CT	TT	C	T
Bai Ku Yao	598	359 (60.0)	219 (36.6)	20 (3.4)	937 (78.3)	259 (21.7)
Han Chinese	585	317 (54.2)	228 (39.0)	40 (6.8)	862 (73.7)	308 (26.3)
*χ*^2^	–	9.316			7.076	
*P*	–	0.009			0.008	
Bai Ku Yao						
Male	286	176 (61.5)	99 (34.6)	11 (3.8)	451 (78.8)	121 (21.2)
Female	312	183 (58.7)	120 (38.5)	9 (2.9)	486 (77.9)	138 (22.1)
*χ*^*2*^	–	1.222			0.163	
*P*	–	0.543			0.687	
Han Chinese						
Male	277	145 (52.3)	114 (41.2)	18 (6.5)	404 (72.9)	150 (27.1)
Female	308	172 (55.8)	114 (37.0)	22 (7.1)	458 (74.4)	158 (25.6)
*χ*^*2*^	–	1.060			0.306	
*P*	–	0.589			0.580	

### Results of sequencing

**Figure 3:**
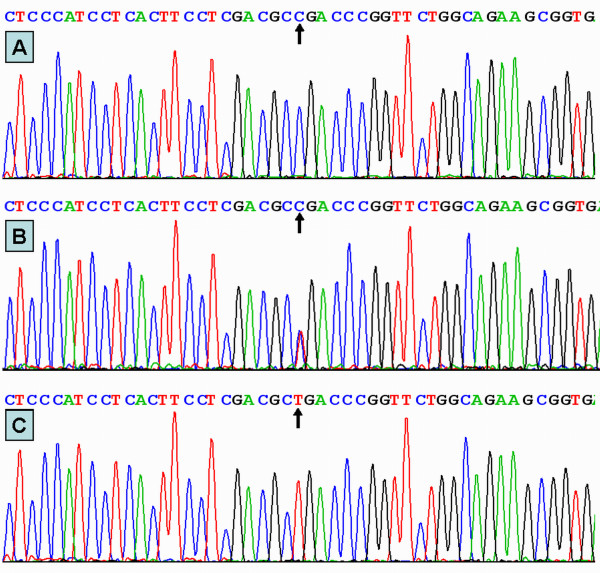
**A part of the nucleotide sequence of rs5888 SNP in the SCARB1 gene**. (A) CC genotype; (B) CT genotype; (C) TT genotype

The results were shown as CC, CT and TT genotypes by PCR-RFLP, the CC, CT and TT genotypes were also confirmed by sequencing (Figure 3); respectively.

### Genotypes and serum lipid levels

As shown in Table 3, the levels of HDL-C and ApoAI in the Bai Ku Yao and Han subjects were different among the three genotypes (*P* < 0.05 for all), the subjects with TT genotype had lower serum HDL-C and ApoAI levels than the subjects with CT or CC genotype. When analysis of covariance was stratified according to sex in both ethnic groups, we found that the subjects with TT genotype in Bai Ku Yao had lower HDL-C and ApoAI levels in males than the subjects with CC or CT genotype (*P* < 0.05 for all), and the T allele carriers had higher TC, LDL-C and ApoB levels in females than the T allele noncarriers (*P* < 0.05 for all). The participants with TT genotype in Han also had a lower tendency of HDL-C and ApoAI levels in males than the participants with CC or CT genotype, but the difference did not reach statistically significant (*P* = 0.063 and *P* = 0.086; respectively).

**Table 3:** The genotypes of SCARB1 rs5888 SNP and serum lipid levels

Genotype	n	TC (mmol/L)	TG (mmol/L)	HDL-C (mmol/L)	LDL-C (mmol/L)	ApoAI (g/L)	ApoB (g/L)	ApoAI/ApoB
Bai Ku Yao								
CC	359	4.30 ± 0.94	0.98 (0.69)	1.66 ± 0.42^a^	2.54 ± 0.76	1.31 ± 0.32^a^	0.84 ± 0.23	1.68 ± 0.76
CT	219	4.44 ± 0.89	1.02 (0.62)	1.69 ± 0.41^a^	2.64 ± 0.75	1.33 ± 0.33^a^	0.87 ± 0.22	1.67 ± 0.72
TT	20	4.04 ± 0.96	1.10 (0.52)	1.43 ± 0.36	2.42 ± 0.88	1.14 ± 0.27	0.78 ± 0.27	1.66 ± 1.00
*F*	–	2.770	0.388	4.001	1.625	3.878	1.871	0.023
*P*	–	0.063	0.824	0.019	0.198	0.021	0.155	0.977
Male								
CC	176	4.39 ± 1.09	1.18 (0.95)	1.70 ± 0.49^a^	2.54 ± 0.92	1.38 ± 0.37^a^	0.83 ± 0.23	1.82 ± 0.95
CT	99	4.43 ± 1.01	1.05 (0.69)	1.74 ± 0.46^b^	2.55 ± 0.86	1.39 ± 0.40^a^	0.83 ± 0.23	1.86 ± 0.92
TT	11	3.97 ± 1.07	1.25 (0.61)	1.30 ± 0.45	2.35 ± 0.99	1.10 ± 0.35	0.76 ± 0.30	1.74 ± 1.32
*F*	–	0.985	1.822	4.743	0.253	3.769	0.559	0.117
*P*	–	0.375	0.402	0.009	0.776	0.024	0.572	0.889
CC	176	4.39 ± 10.9	1.18 (0.95)	1.70 ± 0.49	2.54 ± 0.92	1.38 ± 0.37	0.83 ± 0.23	1.82 ± 0.95
CT + TT	110	4.38 ± 1.02	1.06(0.65)	1.70 ± 0.47	2.53 ± 0.87	1.36 ± 0.40	0.82 ± 0.24	1.85 ± 0.96
*F*	–	0.003	−1.246	0.024	0.014	0.222	0.106	0.072
*P*	–	0.959	0.213	0.877	0.907	0.638	0.745	0.788
Female								
CC	183	4.22 ± 0.76	0.90 (0.51)	1.63 ± 0.33	2.53 ± 0.58	1.24 ± 0.25	0.84 ± 0.22	1.56 ± 0.48
CT	120	4.45 ± 0.78^c^	1.00 (0.61)	1.65 ± 0.35	2.72 ± 0.64^c^	1.27 ± 0.25	0.90 ± 0.19	1.49 ± 0.44
TT	9	4.19 ± 0.84	1.05 (0.38)	1.51 ± 0.24	2.57 ± 0.74	1.16 ± 0.16	0.84 ± 0.24	1.46 ± 0.31
*F*	–	3.562	2.102	0.655	3.881	1.198	2.601	1.121
*P*	–	0.030	0.350	0.520`	0.022	0.303	0.076	0.327
CC	183	4.22 ± 0.76	0.90 (0.51)	1.63 ± 0.33	2.53 ± 0.58	1.24 ± 0.25	0.84 ± 0.22	1.56 ± 0.48
CT + TT	129	4.43 ± 0.78	1.01(0.60)	1.64 ± 0.34	2.71 ± 0.65	1.26 ± 0.24	0.89 ± 0.20	1.48 ± 0.44
*F*	–	6.103	−1.429	0.054	7.234	0.542	4.473	2.224
*P*	–	0.014	0.153	0.817	0.008	0.462	0.035	0.137
Han Chinese								
CC	317	4.73 ± 0.96	0.97 (0.82)	1.76 ± 0.49^a^	2.75 ± 0.76	1.36 ± 0.31^a^	0.82 ± 0.19	1.74 ± 0.54
CT	228	4.71 ± 1.01	1.05 (0.78)	1.72 ± 0.44^a^	2.73 ± 0.85	1.35 ± 0.31^a^	0.82 ± 0.21	1.74 ± 0.54
TT	40	4.54 ± 1.05	0.99 (0.97)	1.55 ± 0.35	2.58 ± 0.67	1.23 ± 0.21	0.81 ± 0.18	1.56 ± 0.35
*F*	–	0.821	1.764	4.125	0.965	3.824	0.044	2.313
*P*	–	0.441	0.414	0.017	0.381	0.022	0.957	0.100
Male								
CC	145	4.73 ± 0.94	1.11 (0.89)	1.68 ± 0.54	2.74 ± 0.72	1.34 ± 0.36	0.83 ± 0.18	1.67 ± 0.50
CT	114	4.70 ± 1.07	1.13 (0.90)	1.64 ± 0.39	2.74 ± 0.91	1.32 ± 0.32	0.84 ± 0.23	1.67 ± 0.54
TT	18	4.59 ± 1.22	1.23 (1.79)	1.43 ± 0.26	2.47 ± 0.65	1.18 ± 0.18	0.83 ± 0.20	1.49 ± 0.37
*F*	–	0.233	1.500	2.793	1.070	2.474	0.036	1.160
*P*	–	0.793	0.472	0.063	0.344	0.086	0.965	0.315
CC	145	4.73 ± 0.94	1.11 (0.89)	1.68 ± 0.54	2.74 ± 0.72	1.34 ± 0.36	0.83 ± 0.18	1.67 ± 0.50
CT + TT	132	4.69 ± 1.09	1.15(0.94)	1.61 ± 0.38	2.71 ± 0.88	1.30 ± 0.30	0.84 ± 0.22	1.65 ± 0.52
*F*	–	0.212	−1.071	1.746	0.139	1.216	0.041	0.124
*P*	–	0.646	0.284	0.187	0.709	0.271	0.839	0.725
Female								
CC	172	4.74 ± 0.98	0.91 (0.80)	1.83 ± 0.43	2.76 ± 0.80	1.38 ± 0.27	0.81 ± 0.19	1.80 ± 0.57
CT	114	4.69 ± 0.94	0.96 (0.60)	1.78 ± 0.48	2.70 ± 0.78	1.37 ± 0.31	0.80 ± 0.19	1.80 ± 0.53
TT	22	4.54 ± 0.91	0.94 (0.69)	1.68 ± 0.38	2.68 ± 0.69	1.29 ± 0.23	0.80 ± 0.15	1.64 ± 0.34
*F*	–	0.559	0.914	1.343	0.373	1.139	0.115	0.937
*P*	–	0.572	0.633	0.263	0.689	0.322	0.891	0.393
CC	172	4.74 ± 0.98	0.91 (0.80)	1.83 ± 0.43	2.76 ± 0.80	1.38 ± 0.27	0.81 ± 0.19	1.80 ± 0.57
CT + TT	136	4.66 ± 0.93	0.96(0.60)	1.76 ± 0.46	2.69 ± 0.77	1.36 ± 0.30	0.80 ± 0.18	1.77 ± 0.50
*F*		0.644	−0.657	1.841	0.730	0.602	0.227	0.255
*P*		0.423	0.511	0.176	0.394	0.439	0.634	0.614

TC, total cholesterol; TG, triglyceride; HDL-C, high-density lipoprotein cholesterol; LDL-C, low-density lipoprotein cholesterol; ApoAI, apolipoprotein AI; ApoB, apolipoprotein B. The value of TG was presented as median (interquartile range), the difference among the genotypes was determined by the Kruskal-Wallis test or the Wilcoxon-Mann–Whitney test. ^a^*P* <0.05, ^b^*P* <0.01 in comparison with the TT genotype of the same ethnic group and ^c^*P* <0.05 in comparison with the CC genotype of the same ethnic group.

### Relative factors for serum lipid parameters

Multiple linear regression analysis showed that serum HDL-C and ApoAI levels were correlated with genotypes in the combined populations of Bai Ku Yao and Han, Bai Ku Yao, and Han; respectively (*P* < 0.05 for all). When multiple linear regression analysis was performed according to sex in both ethnic groups, we found that the levels of HDL-C and ApoAI in Bai Ku Yao were correlated with genotypes in males (*P* < 0.05 for each, Table 4) but not in females. The levels of TC and LDL-C in Bai Ku Yao were also associated with genotypes in females but not in males. The levels of TG and HDL-C in Han were correlated with genotypes in males (*P* < 0.05 for each). Serum lipid parameters were also correlated with sex, age, BMI, alcohol consumption, cigarette smoking, and blood pressure in both ethnic groups (Tables 5 and 4).

**Table 4:** Correlative factors for serum lipid parameters between males and females

Lipid parameter	Relative factor	Unstandardized coefficient	Std. error	Standardized coefficient	*t*	*P*
Bai Ku Yao/male						
TC	Body mass index	0.120	0.028	0.245	4.268	0.000
TG	Body mass index	0.112	0.033	0.200	3.436	0.001
HDL-C	Alcohol consumption	0.192	0.036	0.304	5.274	0.000
	Age	0.006	0.002	0.189	3.302	0.001
	Genotype	−0.416	0.137	−0.166	−3.036	0.003
	Body mass index	−0.026	0.012	−0.116	−2.116	0.035
LDL-C	Body mass index	0.098	0.024	0.236	4.092	0.000
ApoAI	Alcohol consumption	0.170	0.028	0.342	6.048	0.000
	Age	0.004	0.001	0.155	2.743	0.006
	Genotype	−0.287	0.107	−0.145	−2.690	0.008
ApoB	Body mass index	0.029	0.006	0.267	4.677	0.000
ApoAI/ApoB	Alcohol consumption	0.376	0.071	0.301	5.274	0.000
	Body mass index	−0.069	0.025	−0.157	−2.752	0.006
Bai Ku Yao/female						
TC	Systolic blood pressure	0.007	0.003	0.145	2.602	0.010
	Body mass index	0.036	0.017	0.120	2.138	0.033
	Genotype	0.165	0.078	0.118	2.118	0.035
TG	Alcohol consumption	0.311	0.079	0.219	3.961	0.000
LDL-C	Genotype	0.151	0.061	0.135	2.465	0.014
	Body mass index	0.034	0.014	0.141	2.519	0.012
	Age	0.005	0.003	0.122	1.997	0.047
ApoAI	Age	0.002	0.001	0.141	2.509	0.013
ApoB	Systolic blood pressure	0.002	0.001	0.175	3.134	0.002
	Body mass index	0.012	0.005	0.143	2.572	0.011
ApoAI/ApoB	Diastolic blood pressure	−0.007	0.003	−0.139	−2.466	0.014
Han/male						
TC	Diastolic blood pressure	0.019	0.005	0.227	3.783	0.000
	Age	0.013	0.003	0.239	4.243	0.000
	Alcohol consumption	0.249	0.071	0.186	3.494	0.001
	Body mass index	0.031	0.015	0.120	2.108	0.036
TG	Body mass index	0.119	0.021	0.320	5.729	0.000
	Cigarette smoking	0.282	0.092	0.172	3.069	0.002
	Genotype	0.292	0.132	0.125	2.216	0.027
HDL-C	Alcohol consumption	0.177	0.034	0.287	5.223	0.000
	Age	0.006	0.001	0.237	4.082	0.000
	Body mass index	−0.029	0.007	−0.243	−4.147	0.000
	Diastolic blood pressure	0.006	0.002	0.156	2.523	0.012
	Genotype	−0.082	0.041	−0.108	−2.011	0.045
LDL-C	Age	0.009	0.003	0.210	3.450	0.001
	Diastolic blood pressure	0.009	0.004	0.142	2.181	0.030
	Body mass index	0.026	0.013	0.128	2.082	0.038
ApoAI	Alcohol consumption	0.161	0.023	0.369	6.960	0.000
	Age	0.005	0.001	0.281	5.301	0.000
ApoB	Body mass index	0.015	0.003	0.283	5.122	0.000
	Age	0.003	0.001	0.245	4.418	0.000
	Alcohol consumption	0.035	0.015	0.132	2.384	0.018
ApoAI/ApoB	Body mass index	−0.036	0.008	−0.272	−4.721	0.000
	Alcohol consumption	0.161	0.039	0.240	4.150	0.000
Han/female						
TC	Age	0.020	0.004	0.292	5.413	0.000
	Body mass index	0.074	0.017	0.232	4.308	0.000
TG	Body mass index	0.057	0.016	0.196	3.499	0.001
HDL-C	Cigarette smoking	0.479	0.198	0.137	2.425	0.016
LDL-C	Age	0.016	0.003	0.299	5.612	0.000
	Body mass index	0.066	0.014	0.254	4.769	0.000
ApoAI	Age	0.004	0.001	0.209	3.761	0.000
	Cigarette smoking	0.316	0.124	0.142	2.559	0.011
ApoB	Body mass index	0.022	0.003	0.344	6.530	0.000
	Age	0.003	0.001	0.223	4.228	0.000
ApoAI/ApoB	Body mass index	−0.042	0.010	−0.236	−4.335	0.000
	Cigarette smoking	0.883	0.232	0.207	3.810	0.000

TC, total cholesterol; TG, triglyceride; HDL-C, high-density lipoprotein cholesterol; LDL-C, low-density lipoprotein cholesterol; ApoAI, apolipoprotein AI; ApoB, apolipoprotein B.

**Table 5:** Correlative factors for serum lipid parameters

Lipid parameter	Relative factor	Unstandardized coefficient	Std. error	Standardized coefficient	*t*	*P*
Both Yao and Han						
TC	Age	0.011	0.002	0.181	6.402	0.000
	Body mass index	0.059	0.009	0.180	6.419	0.000
	Ethnic group	0.343	0.054	0.177	6.368	0.000
	Diastolic blood pressure	0.010	0.003	0.115	3.850	0.000
	Alcohol consumption	0.089	0.039	0.064	2.285	0.022
TG	Body mass index	0.078	0.010	0.211	7.567	0.000
	Sex	−0.257	0.071	−0.117	−3.631	0.000
	Alcohol consumption	0.207	0.052	0.133	3.981	0.000
	Ethnic group	0.167	0.063	0.076	2.657	0.008
HDL-C	Age	0.005	0.001	0.174	6.163	0.000
	Alcohol consumption	0.172	0.021	0.275	8.177	0.000
	Sex	0.155	0.029	0.176	5.435	0.000
	Ethnic group	0.115	0.025	0.130	4.548	0.000
	Body mass index	−0.018	0.004	−0.119	−4.252	0.000
	Genotype	−0.049	0.020	−0.066	−2.389	0.017
LDL-C	Body mass index	0.051	0.008	0.193	6.678	0.000
	Age	0.008	0.001	0.167	5.731	0.000
	Ethnic group	0.103	0.045	0.066	2.313	0.021
	Diastolic blood pressure	0.007	0.002	0.089	2.894	0.004
	Alcohol consumption	−0.065	0.032	−0.059	−2.029	0.043
ApoAI	Alcohol consumption	0.156	0.015	0.346	10.638	0.000
	Age	0.004	0.001	0.194	7.111	0.000
	Ethnic group	0.086	0.018	0.136	4.852	0.000
	Genotype	−0.132	0.039	−0.092	−3.420	0.001
	Sex	0.065	0.020	0.103	3.285	0.001
ApoB	Body mass index	0.018	0.002	0.249	8.686	0.000
	Age	0.002	0.000	0.148	5.150	0.000
	Ethnic group	−0.038	0.012	−0.089	−3.237	0.001
	Diastolic blood pressure	0.002	0.001	0.080	2.669	0.008
ApoAI/ApoB	Alcohol consumption	0.267	0.031	0.286	8.523	0.000
	Body mass index	−0.042	0.006	−0.190	−6.777	0.000
	Ethnic group	0.142	0.038	0.108	3.755	0.000
	Sex	0.105	0.043	0.080	2.458	0.014
Bai Ku Yao						
TC	Body mass index	0.067	0.016	0.172	4.214	0.000
	Age	0.007	0.002	0.109	2.655	0.008
	Diastolic blood pressure	0.008	0.004	0.083	1.972	0.049
TG	Alcohol consumption	0.258	0.067	0.198	3.872	0.000
	Body mass index	0.054	0.016	0.132	3.302	0.001
	Sex	−0.330	0.106	−0.169	−3.116	0.002
	Cigarette smoking	−0.153	0.070	−0.120	−2.185	0.029
HDL-C	Alcohol consumption	0.126	0.022	0.228	5.777	0.000
	Age	0.004	0.001	0.142	3.586	0.000
	Genotype	−0.245	0.090	−0.106	−2.714	0.007
LDL-C	Body mass index	0.062	0.013	0.194	4.855	0.000
	Age	0.005	0.002	0.098	2.437	0.015
ApoAI	Alcohol consumption	0.143	0.016	0.333	8.708	0.000
	Age	0.003	0.001	0.140	3.655	0.000
	Genotype	−0.177	0.068	−0.099	−2.611	0.009
ApoB	Body mass index	0.019	0.004	0.205	5.064	0.000
	Age	0.002	0.001	0.104	2.564	0.011
	Alcohol consumption	−0.032	0.012	−0.107	−2.611	0.009
	Diastolic blood pressure	0.002	0.001	0.097	2.306	0.021
ApoAI/ApoB	Alcohol consumption	0.311	0.039	0.309	7.940	0.000
	Body mass index	−0.044	0.012	−0.137	−3.522	0.000
Han Chinese						
TC	Age	0.016	0.002	0.260	6.443	0.000
	Body mass index	0.050	0.011	0.174	4.374	0.000
	Diastolic blood pressure	0.012	0.004	0.142	3.341	0.001
	Alcohol consumption	0.164	0.061	0.101	2.697	0.007
TG	Body mass index	0.094	0.013	0.270	6.949	0.000
	Cigarette smoking	0.357	0.063	0.221	5.697	0.000
HDL-C	Age	0.006	0.001	0.219	5.533	0.000
	Sex	0.244	0.040	0.264	6.074	0.000
	Alcohol consumption	0.190	0.033	0.250	5.702	0.000
	Body mass index	−0.022	0.005	−0.163	−4.119	0.000
	Genotype	−0.075	0.028	−0.101	−2.635	0.009
LDL-C	Age	0.012	0.002	0.245	5.873	0.000
	Body mass index	0.043	0.009	0.186	4.533	0.000
	Diastolic blood pressure	0.006	0.003	0.090	2.062	0.040
ApoAI	Age	0.005	0.001	0.251	6.600	0.000
	Alcohol consumption	0.165	0.022	0.326	7.621	0.000
	Sex	0.134	0.026	0.217	5.096	0.000
	Genotype	−0.037	0.019	−0.074	−1.979	0.048
ApoB	Body mass index	0.018	0.002	0.323	8.524	0.000
	Age	0.003	0.000	0.228	5.967	0.000
	Cigarette smoking	0.028	0.010	0.105	2.807	0.005
ApoAI/ApoB	Body mass index	−0.039	0.006	−0.256	−6.450	0.000
	Sex	0.232	0.047	0.218	4.931	0.000
	Alcohol consumption	0.184	0.039	0.211	4.724	0.000

TC, total cholesterol; TG, triglyceride; HDL-C, high-density lipoprotein cholesterol; LDL-C, low-density lipoprotein cholesterol; ApoAI, apolipoprotein AI; ApoB, apolipoprotein B.

## Discussion

The current study shows that the levels of serum TC, HDL-C, LDL-C, ApoAI were lower, whereas the levels of ApoB was higher in Bai Ku Yao than in Han. There was no significant difference in the levels of TG and the ratio of ApoAI to ApoB between the two ethnic groups. It is well known that dyslipidemia is a complex trait caused by both environmental and genetic factors. Bai Ku Yao is an isolated subgroup of the Yao minority in China. Strict intra-ethnic marriages have been performed in this population from time immemorial. Therefore, we are confident that the hereditary characteristics and genotypes of some lipid metabolism-related genes in this population may be different from those in Han Chinese.

In the present study, we showed that there was significant difference in the allelic and genotypic frequencies of SCARB1 rs5888 SNP between the two ethnic groups. The frequencies of CC genotypes were higher in Bai Ku Yao than in Han. The frequency of C allele was also higher in Bai Ku Yao than in Han (78.3% vs 73.7%, *P* < 0.01). To the best of our knowledge, this is the first study to report the association of SCARB1 rs5888 SNP and serum lipid levels in the Bai Ku Yao population. The genotypic and allelic frequency of SCARB1 rs5888 SNP in Han population observed in our research was also consistent with the frequency of Han Chinese from Beijing published on NCBI database (http://www.ncbi.nlm.nih.gov/projects/SNP/snp_ref.cgi?rs=5888). Several similar researches have carried out in European and North American populations. In a initial study in Spain population, Acton *et al.*[20] determined the polymorphism of Exon 8 (C/T) (rs5888) and showed that the frequency of C allele was 56.22% in a general white Spanish population. In another study, McCarthy *et al.*[22] reported the frequency of C allele was 44.7% in Israeli, 60.2% in Finn and 52.5% in Swede; respectively. In an American population of Central European ancestry, the allele frequency of C in the SCARB1 gene was 59.0% [19]. In a population of Caucasian in America, the frequency of C allele of rs5888 polymorphism in the SCARB1 gene was 51.39% in men and women [23]. In Korean subjects, however, the frequency of the common C allele of SCARB1 *Hae*III (rs5888) was 67% in control and 83% in CHD [24] which was similar to our study in Chinese population. Thus, our findings, coupled with reports in the literature, provide evidence for the prevalence of the C allele variation of rs5888 in the SCARB1 gene may have an ethnic specificity.

Several ethnic distinct populations have been previously reported that the T allele of SCARB1 rs5888 is correlated with an increase of serum HDL-C concentration and a decrease of LDL-C concentration. In a population of healthy Spanish, Acton *et al.*[20] reported that the T allele carriers have significant lower concentration of LDL-C than the T allele noncarriers in females but not in males. They did not find the association of exons 8 (rs5888) variant in SCARB1 with HDL-C levels. In a population of Caucasian in North America, Osgood *et al.*[23] showed that the T allele was associated with an increase in HDL particle size in men and women, serum HDL-C levels in men, and a decrease in LDL-C levels in women. In a cohort of the Amish Family Diabetes, Roberts *et al.*[19] documented that the rs5888 variant was significantly associated with higher HDL-C level in women younger than 50 years but not in women aged 50 years or older. In a case–control study including 137 subject with CHD and 124 age-matched controls, the concentrations of plasma HDL-C and ApoAI varied significantly among *Hae*III (rs5888) genotypes in the CHD patients but not in the controls, the common genotype (CC) was associated with a lower concentrations of plasma HDL-C and ApoAI [24]. Morabia *et al.*[21] also found that the T allele has higher levels of HDL-C and HDL-C/LDL-C ratio in men. Two other studies carried out by McCarthy *et al.*[20,30] did not find the single rs5888 variant was correlated with lipid profiles, when combined with other polymorphisms in the SCARB1 gene, the combined genotypes were associated with TG, TG/HDL-C ratio and HDL-C in the women. However, some researches found that the rs5888 T allele was associated with increased levels of TC, LDL-C and TG. In a research on subjects with heterozygous familial hypercholesterolemia, Tai *et al.*[31] found that the exon 8 (rs5888) polymorphism was associated with increased levels of TC, VLDL-C, LDL-C and TG. Morabia *et al.*[21] also showed that the genotype of TT had higher levels of TC and LDL-C than the CC genotype in females.

It is well documented that an overexpression of SCARB1 in the animal model could induce a decreased in the levels of HDL-C, LDL-C, and ApoB [10,15] and mice with attenuated expression of SCARB1 display elevated concentrations of LDL-C as well as HDL-C [11,12]. In human, a novel 11-base pair deletion mutation in the promoter region of the SCARB1 gene explained some of the interindividual variation in plasma HDL-C levels in Chinese Taiwanese [32]. A study of women with hyperalphalipoproteinemia also showed that SCARB1 protein was inversely correlated with HDL-C levels and HDL size [33], which suggesting that the SCARB1 in human may partly play the similar role as in the animals. Furthermore, a recent research reported that the rs5888 variant in the SCARB1 affected SCARB1 RNA secondary structure, protein translation, and was significantly associated with reduced SCARB1 protein expression and function *in vitro*[34]. In the present research, however, we showed that the subjects with TT genotype in Bai Ku Yao had lower HDL-C and ApoAI levels in males than the subjects with CC or CT genotype, and the T allele carriers had higher TC, LDL-C and ApoB levels in females than the T allele noncarriers. The participants with TT genotype in Han also had a lower tendency of HDL-C and ApoAI levels in males than the participants with CC or CT genotype, but the difference did not reach statistically significant. The association of serum HDL-C and ApoAI levels and genotypes was confirmed by the multiple linear regression analysis in both ethnic groups. It is difficult to explain these contradictory findings. The possible reasons may be as follows. Firstly, in our study the frequency of C allele was 78.3% in Bai Ku Yao and 73.7% in Han. In white populations, however, the C allelic frequency of SCARB1 rs5888 was varied from 40% to 60%, which suggests that our study populations had different genetic background from white populations. Moreover, the C-T change at exon 8 did not affect the amino acid sequence of the protein, the SNP might be in linkage disequilibrium with a functional mutation in the SCARB1 gene, or alternatively with another nearby functional variant at the chromosomal region of 12q24 where several other candidate genes involved in lipid metabolism have been localized (i.e. ACACB, PLA2, CLTA, MVK-MMAB, ACADS, and TCF1) [20,23]. Thus, our findings contradict with previous papers may attribute to different functional mutation of the SCARB1 rs5888 or to be in linkage disequibibrium with other SNPs. Secondly, it might because of difference in study designs, sample size, as well as gene-enviromental interactions. Lastly, the size of our study population is a bit small, and the number of subjects with TT genotype in both ethnic groups is also small, which might not have had the power to detect the association of TT genotype and serum lipid levels.

In addition to the influence of genetic factors, environmental factors also play an important role in determining serum lipid levels [25,26] . In the present study, we also found that serum lipid parameters were correlated with several environmental factors. Although Bai Ku Yao and Han reside in the same region, the diet and lifestyle were significant difference between the two ethnic groups. For the Bai Ku Yao population, corn was the staple food and rice, soy, buckwheat, sweet potato, and pumpkin products were the subsidiary foods. Approximately 90% of the beverages were corn wine and rum. The alcohol content is about 15% (v/v). They are also accustomed to drink Hempseed soup. In contrast, rice was the staple food and corn, broomcorn, potato, and taro products were the subsidiary foods in the Han population. About 90% of the beverage was rice wine. The content of alcohol is about 30% (v/v). These differences in diet and lifestyle could partly account for the differences in serum lipid profiles between the two ethnic groups. Corn contains abundant dietary fiber and plant protein [35]. Consumption of dietary fiber, specifically the soluble type, such as pectins and guar gum can decrease serum TC levels [36,37]. Plant protein might promote the transportation and excretion of free cholesterol. Dietary soy protein has well-documented beneficial effects on serum lipid concentrations [38,39]. Buckwheat protein products have a potent hypocholesterolemic activity [40,41]. Ludvik *et al.*[42] found that ingestion of 4 g/day caiapo (the extract of white-skinned sweet potato) for 6 weeks reduced TC and LDL-C in type 2 diabetic patients previously treated by diet alone. Studies have demonstrated that pumpkin is a useful therapy for hypercholesterolemia through reducing oxidative stress and cholesterol levels [43]. There are more than 29 fat soluble constituents in Hempseed, among which saturated and unsaturated fatty acid methyl esters account for 12.36% and 86.96%; respectively [44]. Previous experimental and clinical studies have demonstrated that Hempseed or Hempseed oil can decrease TC, TG and LDL-C levels, reduce atherogenic index, and increase HDL-C concentration [25,26,45].

## Conclusion

The present study shows that the frequency of T allele of SCARB1 rs5888 SNP is lower in Bai Ku Yao than in Han Chinese. The subjects with TT genotype in Bai Ku Yao had lower HDL-C and ApoAI levels in males than the subjects with CC or CT genotype, and the T allele carriers had higher TC, LDL-C and ApoB levels in females than the T allele noncarriers. The participants with TT genotype in Han also had a lower tendency of HDL-C and ApoAI levels in males than the participants with CC or CT genotype, but the difference did not reach statistically significant. The levels of HDL-C and ApoAI were correlated with genotypes in the both ethnic groups. These results suggest that the differences in serum lipid levels between the two enthnic groups might partially attribute to the differences in the SCARB1 rs5888 SNP and several environmental factors.

## Competing interests

The authors declare that they have no competing interests.

## Authors’ contributions

DFW participated in the design, undertook genotyping, and drafted the manuscript. RXY conceived the study, participated in the design, carried out the epidemiological survey, collected the samples, and helped to draft the manuscript. XJH, LHHA, XLC, LM, QL and TTY collaborated to the genotyping. JZW and SLP carried out the epidemiological survey and collected the samples. All authors read and approved the final manuscript.
